# Social isolation and depression as risk factors for weight loss of 5kg or more among older Korean adults

**DOI:** 10.1371/journal.pone.0299096

**Published:** 2024-03-13

**Authors:** Youngjoon Bae, Mark C. Pachucki

**Affiliations:** 1 Center on Aging and Population Sciences, The University of Texas at Austin, Austin, Texas, United States of America; 2 Department of Sociology, University of Massachusetts Amherst, Amherst, Massachusetts, United States of America; Emory University, School of Public Health, UNITED STATES

## Abstract

Given a well-known overlapping prevalence of social isolation with loneliness and depression among older adults, this study aimed to contextually investigate the relationship of these constructs with weight loss of more than 5kg in a year, with a special focus on the intersection of living alone and marital dissolution as key dimensions of isolation. The data were obtained from the Korean Longitudinal Study of Aging (KLoSA) from 2006, 2008, 2010, 2012, 2014, 2016, and 2018, with an adult sample of those aged 65 and older (n = 5,481). The study evaluated several critical dimensions of social isolation: living alone, transition to living alone, infrequent social contact with children or friends, and infrequent social participation. These dimensions were examined individually and as a composite scale, along with loneliness and depressive symptoms, to determine their association with weight loss of 5kg or greater among older men and women. Generalized Estimating Equation (GEE) regression models enabled investigation of whether socially isolated men and women tended to lose 5kg or more in weight, given other confounding factors. Surprisingly, the results showed no evidence of such a trend. However, significant associations were found between weight loss and changes in living alone and marital status. For older men, transitioning to living alone without a change in marital status was linked to significant weight loss. For older women, transitioning to living alone following widowhood or divorce was the risk factor. These relationships remained significant even after adjusting for depression and a wide range of covariates. Additional analysis testing a cumulative effect revealed that only depression was a risk factor for being underweight at the last observation. Therefore, to prevent a clinically risky extent of weight loss, health policies for older Koreans should focus on those who transition to living alone, especially due to spousal bereavement or divorce (among women) and separation from living with children (among men).

## Introduction

Social isolation has been identified as a risk factor for mortality according to various studies [1–3]. However, the exact reason behind this phenomenon is not yet fully understood. One possible explanation is that increased social isolation among older adults may lead to weight loss, which is a severe concern that may contribute to premature death. Studies have shown a link between weight loss and higher mortality risk [4–7]. Despite this, no conclusive evidence has confirmed how social isolation leads to weight loss.

Social isolation in later life can be largely attributed to living alone, often due to spousal bereavement. Studies have shown that both living alone and spousal bereavement are related to weight loss [8,9]. However, it is not clear how these relationships are altered when both aspects are considered concurrently and rigorously tested. In previous research on social isolation, one of these factors was usually studied, but not both [10–12]. It is worth noting that in South Korea (hereafter Korea), widows/widowers do not necessarily live alone, and older adults making the transition to living alone may not have necessarily experienced spousal bereavement recently. Many widows/widowers live with their children after the loss of their spouse, while others may choose to live alone for more independence. In other words, living alone and widowhood are conceptually and practically distinct, and these statuses may be associated with weight loss in different ways.

The link between living alone or being a widow/er with weight loss may be significantly influenced by loneliness and depression. Loneliness is commonly associated with living alone [13], while poor health outcomes during widowhood can be attributed to depression, as the death of a spouse is considered one of the most stressful life events [14]. Additionally, depression has been found to be a factor in subjective aspects of social isolation [15] and weight change [16]. By taking into account loneliness and depression, it is possible to better understand the effects of social isolation in living alone and widowhood/spousal bereavement.

The primary aim of this study is to examine how social isolation among older men and women is prospectively associated with a clinically dangerous level of weight loss (5kg or greater loss). The next purpose is to adjudicate the relative contributions of living alone and widowhood, as components of social isolation, to dangerous weight loss. To achieve this, the study compares various pathways at the intersection of living alone and spousal bereavement, while accounting for loneliness and depression, in relation to weight loss.

This study is unique in that it investigates a clinically dangerous amount of weight loss in the context of multiple dimensions of social isolation such as living alone, widowhood, transition to living alone due to spousal bereavement or other circumstances, and social contact in a large population sample. Furthermore, this study is rare as it follows a sample over time prospectively.

### Why does weight loss matter among older adults?

Several studies have shown that losing body weight is a risk factor for premature death [4,5], especially in older men [17]. Significant weight loss, such as losing 5kg or more, is a common sign of older adult frailty [18] and is also linked to mortality [7]. Additionally, if the weight loss is greater than 5% of body weight, even "intentional" weight loss is considered clinically risky for older adults [19]. In older populations, adults tend to experience a natural decrease in body weight due to two physiological changes: sarcopenia (a loss of muscle mass) and anorexia (a decline in appetite) [20,21]. At the extremes, these physiological changes can lead to clinically risky levels of weight loss (considered to be more than 5kg, or at least 5% of one’s body weight over a 5–10 year period) [4]. Given that 15% to 20% of older adults experience risky weight loss [4], it is important to pay close attention to older adults’ changes in weight.

### Living alone and weight loss

While social isolation has been linked to poor nutrition and a reduced likelihood of being overweight or obese [10,22,23], its impact on weight loss is still unclear. On the other hand, living alone, going through a divorce, and feeling disconnected from others (all components of social isolation [2,24–26]) as well as loneliness and depression (which can be confounding factors) have documented relationships with body weight changes.

Research on the effects of living alone on weight change in different national contexts has shown inconsistent results, perhaps due to social and cultural variation. For example, a study conducted on older South Korean men found that those living alone tend to lose more weight [27]. However, an analysis of an older Italian sample revealed an inverse association [28]. It is important to consider the possibility of non-linearities in social isolation as well. A study with older Japanese men living alone showed contrasting patterns in weight change [29]. In addition, those who live alone have a higher chance of being underweight or obese. Even though clinically risky weight loss (i.e., in excess of 5kg, or loss of 5% of body weight) is not precisely comparable to weight status (being underweight or obese), these findings imply possible links between living alone, weight reduction, and being underweight. Further, as the correlation between living alone and weight loss is only evident in cross-sectional studies and not in longitudinal studies [9], it is extremely important to examine the possible effects of living alone on weight loss in both the short-term (as a repeated measure) and long-term (as a cumulative measure) with longitudinal data. Prior research suggests that weight loss connected to living alone in the short term may be an indicator of ill health; in the long term, it may be related to mortality [6]. In addition, a repeated measure, which captures short-term living alone, can help determine if participants living alone tend to experience weight loss repeatedly. Meanwhile, a cumulative measure over the longer term can account for a period effect of living alone in weight loss.

For older adults, it is important to consider not only the challenges of living alone but also all of the changes in living arrangements that accompany such a transition. The absence of a caregiver during a transition can make an older adult vulnerable to struggles with physical and mental health, including weight loss. However, the relationship between a transition to living alone and weight loss can be influenced by stressful life experiences like bereavement, divorce, or separation which usually leads to living alone. To the best of our knowledge, there is no study yet that directly examines the relationship between living-alone transitions and weight change. Even the most relevant study suggests that older adults who start living alone may not be at risk of poor diet or weight loss. For example, some older men in the United Kingdom who transitioned to living alone tried to proactively learn cooking skills [30]. Thus, it is vital to pay attention to both living alone as a new status, and the *transition* to living alone.

### Marital status and weight loss

Previous research on the correlation between marital status and body weight has produced mixed results. In the United States, both older men and women who were divorced or widowed tended to experience a slight decrease in BMI [31,32]. However, the decrease in body weight was not significant enough to be considered critical (a drop of 5 kg or more). Conversely, a study among Canadian adults (aged 45–85) showed the opposite relationship: women who were unmarried (widowed, divorced/separated, or single) had a higher risk of obesity compared to those who were married [33]. Losing a spouse due to bereavement, divorce, or separation can lead to social isolation, particularly if the loss occurred recently. Studies have shown that the negative effects of such a loss on health are significant for both men and women [34,35]. Similarly, a review of thirty-four studies found strong evidence that spousal bereavement is associated with weight loss, particularly involuntary weight loss [36]. In fact, marital dissolution can have a greater impact on weight loss than marital status affecting men and women similarly [7]. It bears remembering that underlying such effects on weight loss are the stresses and emotional changes associated with the event of losing a spouse, rather than simply a change in marital status.

Several studies have investigated the relationship between sex/gender, weight loss, and marital dissolution [31,32,37]. However, given that none of these studies have examined the impact of living alone or social relationships on these factors, the findings do not necessarily indicate marital dissolution is more detrimental to weight loss than other types of social isolation.

### Social contact and weight loss

Social disconnection, which involves limited social contact with others, is a common sign of social isolation and is associated with less robust health [24,38]. Still, there remains little research to examine the link between social contact frequency and weight loss among older adults. There may be many reasons for this, ranging from a complex chain of factors linking them, to scant evidence despite valiant efforts to assess such an association, to a low prioritization of this question in the scientific community, among others. Additionally, there are few studies examining the relationship between social contact and frailty (measures of which typically include weight loss [39]) particularly along with consideration of social support. One exception was a study of older Koreans, demonstrating that a lack of social connection and support was associated with an increased likelihood of frailty [40]. Similarly, a study of UK adults showed an indirect relationship between social contact and chronic disease mediated by social support [41].

### Loneliness, depression, and weight loss

Finally, studies of social relationships and isolation have found that perceived isolation, typically measured as loneliness, has a greater impact on health than objective aspects of social isolation such as living alone, widowhood, and infrequent social contact [42]. This is evident in findings from various studies in England reporting a connection between loneliness and frailty [43,44]. In these studies, loneliness was a significant factor even when other indicators of social isolation were used. The distinction between objective social isolation and loneliness is clear [25]. Therefore, this paper limits social isolation to objective social isolation and considers loneliness as a confounding factor in the relationship between social isolation and weight loss.

Although depression is not typically considered an element of social isolation, it is often investigated with loneliness because depression and loneliness tend to be confounded [15,45,46]. Moreover, depression is a robust and consistent risk factor for weight loss, frailty, and underweight [15,49–51]. Research using the Health and Retirement Study reported that depression is associated with weight change– both weight loss and weight gain [16]. One recent study found that the severity of depressive symptoms is the strongest risk factor for frailty among older adults with depression [47]. Moreover, it has been found through various Korean studies that individuals who are underweight tend to exhibit depressive symptoms [48,49]. However, these studies do not establish a direct correlation between depression and weight loss. Furthermore, existing research does not yet offer a contextual analysis of how depression and social isolation might be independently related to weight loss.

### Korean context

We seek to contextually examine these multiple dimensions of social isolation and their relationships with weight loss in excess of 5kg during the past year in older adults. To do so, we focus on a sample of older adults in Korea. This is an ideal sample for a number of reasons. First, the proportion of older Koreans in the population has been steadily increasing from 10.8% in 2010 to 17.5% in 2022, and is projected to reach 40% in 2050 [50]. Additionally, the percentage of single-person households among older Koreans has also been rising, a trend expected to continue. As of 2020, 34.9% of households with household heads aged 65 and older were single-person households, and this figure is expected to increase to 41.1% by 2050 [51].

Older Koreans living alone are considered to be socially isolated and socioeconomically vulnerable [52]. A study conducted from 1960 to 2010 found that the majority of those who live alone are widowed women with the lowest level of education [53]. Additionally, a report from the Korean government revealed that older Koreans living alone experience poverty, weak connection with their children, and malnutrition at higher rates than those living with their spouses or children [54]. This suggests that older Koreans living alone, particularly women, may be at a higher risk of suffering from social isolation and adverse health outcomes. Therefore, this study anticipates that one of the potential health problems for these individuals is weight loss of 5kg or more in a year.

### Hypotheses

**Fig 1:**
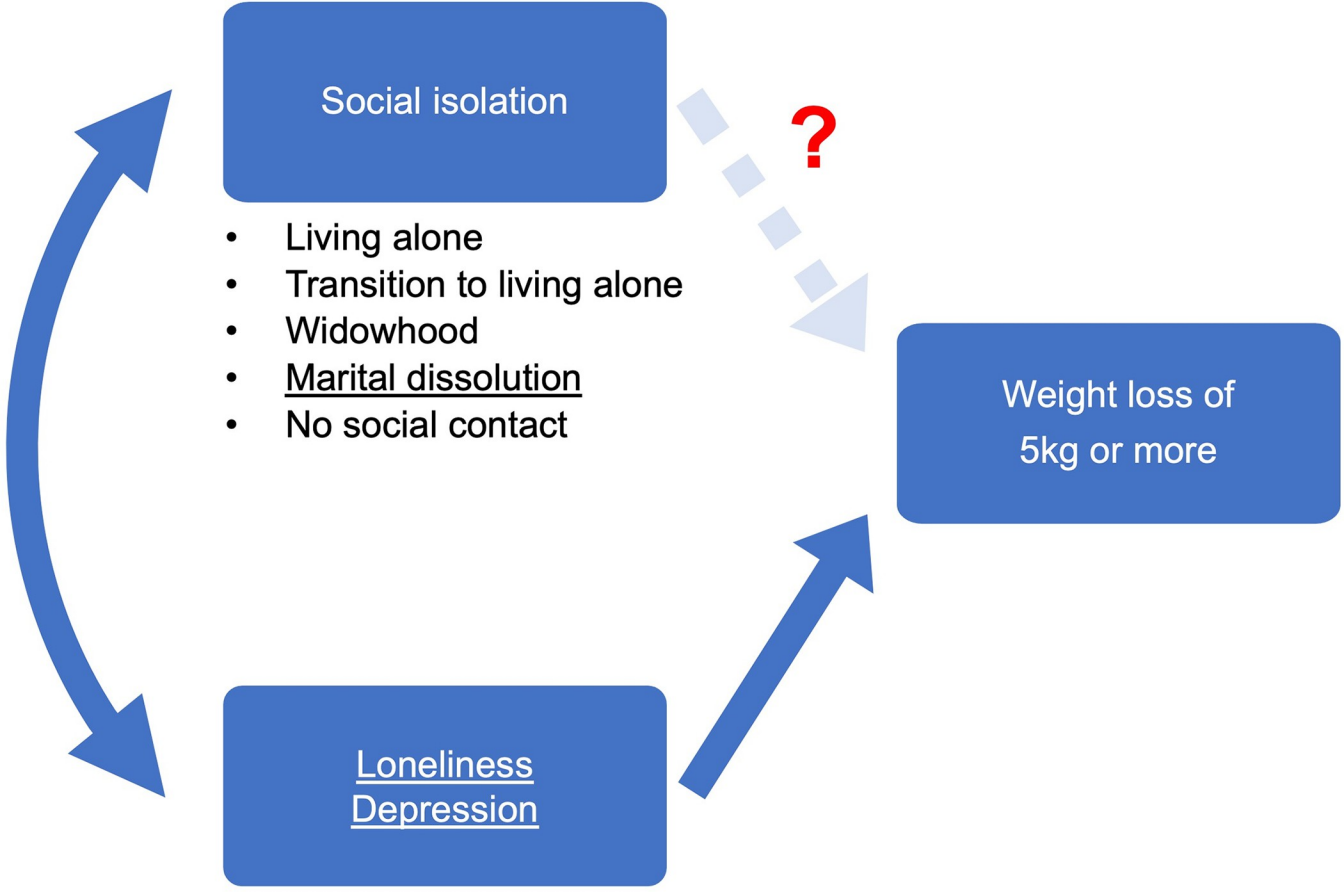
Conceptual model of weight loss of 5kg or more in a year. ^a^ *Note*: Loneliness, depression, and marital dissolution were underlined, given that prior research indicates their associations with ≥5kg weight loss.

**Fig 2:**
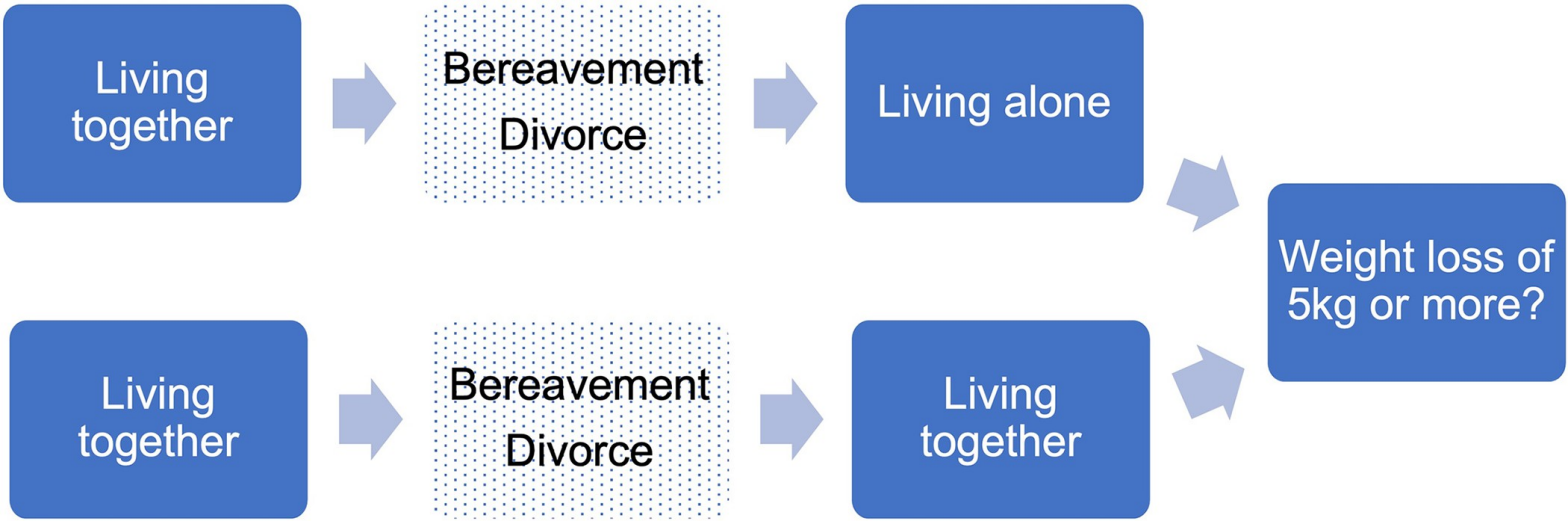
A sub-conceptual model of weight loss of 5kg or more in a year.

To summarize prior research (Fig 1), it can be inferred that loneliness and depression are strong risk factors for experiencing dangerous levels of weight loss. Marital dissolution has also been found to be consistently associated with ≥5kg weight loss, particularly among Korean women living alone. However, this relationship needs to be tested while adjusting for loneliness and depression. Additionally, there is a lack of research on how living alone or together after marital dissolution may be differentially related to weight loss (Fig 2). While social contact appears to have an indirect relationship with weight loss through social support, other aspects of social isolation, such as living alone, widowhood, and transitioning to living alone, have unclear relationships with weight loss. Nevertheless, given the precarious life circumstances of older socially isolated Koreans, social isolation may be associated with ≥5kg weight loss.

Based not only on prior research on social relationships and physiological mechanisms related to weight loss but also on the Korean context, we pose the following hypotheses:

H1: Social isolation increases the risk of 5kg or more weight loss for men and women.
H2: Living alone after spousal bereavement or divorce increases the risk of 5kg or more weight loss for men and women.

## Materials and methods

### Sample

Study data were obtained from the Korean Longitudinal Study of Aging (KLoSA), which is a nationally representative dataset. The survey was conducted every other year, starting in 2006, while using similar questions. The KLoSA website (https://survey.keis.or.kr/eng/klosa/klosa01.jsp) allows for public de-identified data download. Since the KLoSA is a companion to the Health and Retirement Study (HRS), data access instructions are also available on the Gateway to Global Aging Data website (https://g2aging.org/downloads). Also, the minimal data set was provided as S2 Appendix.

We used repeated panels gathered in the years 2006, 2008, 2010, 2012, 2014, 2016, and 2018. These data provide a great deal of contextual information, including demographic background, family ties, health status, employment, financial condition, and quality of life among people over 45 years old. More importantly, these data comprise a rare source simultaneously reporting living arrangements, marital status, social relationships, and change in body weight over 5kg of older Koreans selected from a nationally representative sample. As we utilized publicly accessible de-identified secondary data with no access to a code key, this study did not meet the definition of human subjects research according to the Revised Common Rule (2019) [55], confirmed with the University of Massachusetts at Amherst Human Research Protection Office. The current research is in compliance with the ethical standards of STrengthening the Reporting of OBservational studies in Epidemiology (STROBE).

**Fig 3:**
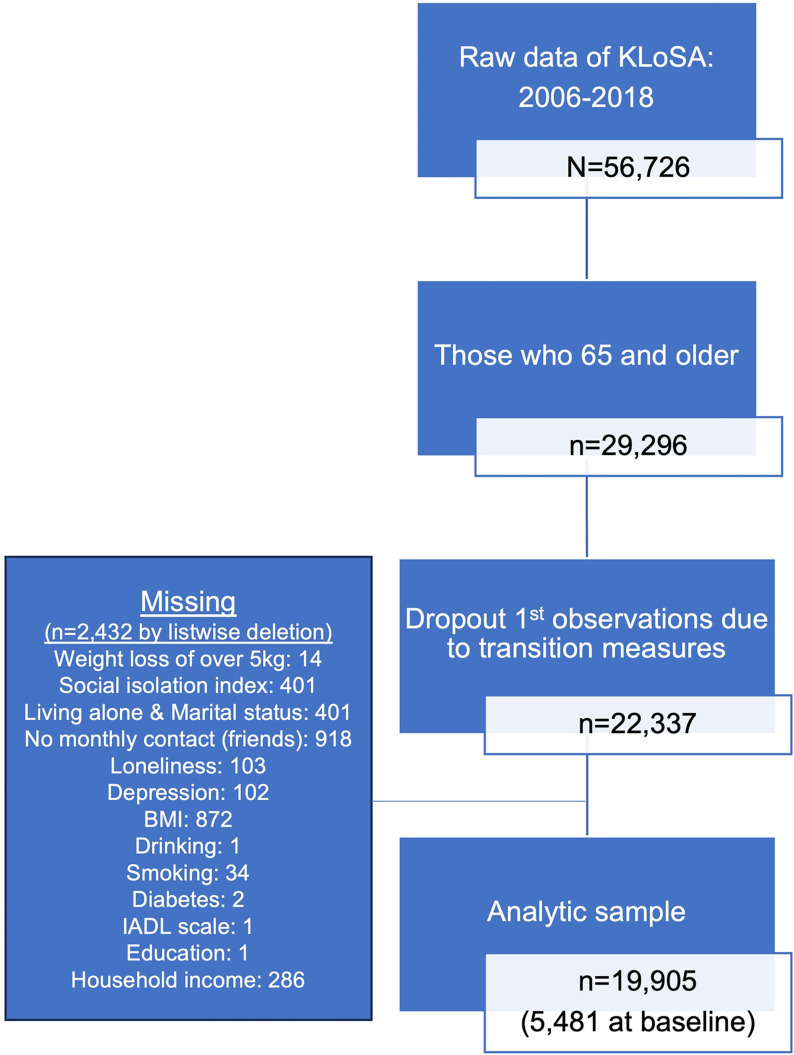
Sample selection process.

The analysis sample involves individuals aged 65 years and above. The frequency of contact with friends and BMI had approximately 4% missing observations. Social isolation and changes in living alone and marital status (measured using a change between waves), had approximately 1.8% missing observations. Household income had 1.3% missing, while other variables had less than 0.2% missing. The final analytic sample used for the study consisted of 19,905 person-year observations from 5,481 participants, including 2,382 men (43%) and 3,099 women (57%). Fig 3 illustrates the sample selection process. During the construction of the composite variable based on living arrangements and marital status, the entry wave information was dropped, leading to intentional data attrition. As a result, longitudinal sample weights were not used.

### Dependent variables

The primary variable of interest is whether the respondent has experienced a weight loss of 5kg or more over the past year. This measure, developed by Fried and colleagues to assess frailty [18], is widely used as a risk factor for mortality and other weight loss measures [4]. The KLoSA survey asked participants if they had lost this amount of weight over the past year. A secondary dependent variable, used only in an ad hoc analysis following the main analyses, is a dichotomous “underweight status” at the final observation. Participants with a BMI below 18.5 were categorized as underweight (otherwise 0).

### Independent variables

The main independent variable is the social isolation index, constructed by adapting the Steptoe Social Isolation Index [56] and the social isolation measure by Kobayashi and Steptoe [10]. The Steptoe index considered marital status, monthly contact with children, other family or friends, and group participation. However, Kobayashi and Steptoe replaced marital status with living alone. This study further adapts the intuition with a novel composite variable to measure different types of transition to living alone depending upon marital status.

The question about living alone was asked in a household roster. Those who responded as living alone were given a code of 1, while those who lived with someone else were given a code of 0. Marital status was re-categorized into three groups: currently married, divorced/separated/never married, and widowed. Using these two measures, we created a variable with seven categories based on changes in living alone and marital status between the previous and current waves. As a result of this process, the first observations of all respondents were automatically excluded. Categories with extremely low numbers of observations were disregarded. The first group was used as a reference. It is important to note that each of these categories is mutually exclusive. The seven categories are listed below, with frequencies and percentages in Table 1:

1. Continued living together without a change in marital status among those who were currently married.
2. Continued living together without a change in marital status among those who were widowed, divorced, or never married (mainly due to children in the households).
3. Continued living alone without a change in marital status (mostly widowed).
4. Transitioned to living alone after spousal bereavement/divorce.
5. Continued living together after spousal bereavement/divorce.
6. Transitioned to living together without a change in marital status.
7. Transitioned to living alone without a change in marital status.

**Table 1:** Frequency of changes in living alone and marital status between waves.

	Person-Year observations	
	Men	Women
1. Continued living together without a change in marital status (married)	7,168 (83.7%)	5,075 (44.8%)
2. Continued living together without a change in marital status (widowed)	250 (2.9%)	2,360 (20.8%)
3. Continued living alone without a change in marital status	332 (3.9%)	1,861 (16.4%)
4. Transitioned to living alone after spousal bereavement/divorce	338 (4.0%)	768 (6.8%)
5. Continued living together after spousal bereavement/divorce	354 (4.1%)	878 (7.7%)
6. Transitioned to living together without a change in marital status	54 (0.6%)	183 (1.6%)
7. Transitioned to living alone without a change in marital status	68 (0.8%)	216 (1.9%)
Total	8,564 (100.0%)	11,341 (100.0%)

Table 1 shows that living alone and widowhood are closely intertwined, and transitions to living alone or with someone else often occur in later life, regardless of changes in marital status. If respondents belonged to categories 3, 4, or 7 (living alone or a transition to living alone), one point was added to their social isolation index given that physical isolation often contributes meaningfully to social isolation. While this may be a strong assumption, we believe it to be justified in the Korean context.

To consider other components of the social isolation index, we included a quantitative aspect of *social disconnectedness*. We looked at three types of social contact: with children, friends, and group activities. The group activities included participation in religious services, senior centers, sports clubs, school reunions, volunteer work, non-governmental organizations, and other activities. We used a threshold of monthly contact [10], and prepared three binary variables: *less than monthly contact with children*, *less than monthly contact with friends*, *and less than monthly group activities*. Each experience was added as one point to the social isolation index, which ranges from 0 to 4. In our analysis, we used the social isolation index and its components exclusively.

### Confounders

Loneliness and depression were considered important confounders and were included in the analysis. They may also be considered to mediate the relationship between social isolation and the outcome variables. However, this study focuses on how social isolation, particularly living alone, is independently related to weight loss regardless of loneliness or depression, probably through life events such as spousal bereavement, divorce, and so on. Thus, we used them as confounders. Many studies have employed this approach [3,10,25,42]. The KLoSA survey asked respondents about their feelings of loneliness and depression over the past week. The survey included questions such as how often the respondents felt alone and how often they felt depressed. The responses were categorized into four choices: very rarely (less than 1 day), sometimes (1–2 days), often (3–4 days), and almost always (5–7 days), with the first category being considered as a reference point. These measures were used to determine the levels of loneliness and depression among the respondents.

In order to investigate the relationship between ≥5kg weight loss and various factors related to nutrition, health, and sociodemographic background, several other variables were included. Nutrition variables are particularly important because they may reveal potential mechanisms of the relationship between social isolation and weight loss. Meanwhile, KLoSA only provides *“number of meals in the prior 2 days (0–6)”* and *“whether participants drink alcohol (1 = respondent drinks)”* regarding nutrition. We assessed both variables and preliminary tests did report significant associations with weight loss among men. Our other study examines the relationship between social isolation and nutrition.

To account for health-related mechanisms and functional capabilities, several variables were included such as body mass index (BMI), type 2 diabetes (1 = currently diagnosed), Instrumental Activities of Daily Living index (IADL), Activities of Daily Living index (ADL), physical activity (1 = exercises at least once a week), and smoking (1 = currently smokes). BMI is calculated by dividing the weight (in kg) by the height (in m^2^) and is a continuous variable. Type 2 diabetes was included because of its relation to nutritional behaviors and frailty [57]. The IADL and ADL indexes are used to measure the availability of independent daily living. A higher score on these indexes indicates greater difficulties in independent living. These indexes also speak to nutrition as IADL includes the ability to prepare meals for oneself, and one of the ADL items indicates the ability to eat food without help.

The study considered various social and demographic factors, including employment status (1 = currently employed), education level (< middle school as reference, middle school, high school, college or higher), geographic location (rural as reference, urban, metropolitan), and annual household income (reported in Won, the monetary unit of Korea, and converted to dollars by multiplying by 1,000). The study used categorical age instead of a continuous variable, which is more appropriate given the non-linear relationship between age and health (age groups = 65–74, reference 75–84, and 85 and older). Finally, time trends were measured by treating years of a wave variable as dummies.

### Analysis strategy

Descriptive statistics were calculated separately for men and women, as well as for those who live alone and those who live with someone else. Means and standard deviations were presented for continuous variables, with percentages for categorical variables. To compare characteristics of those living alone and their counterparts, t-tests were used for continuous variables, and χ^2^ tests for categorical variables.

We utilized generalized estimating equation (GEE) models, which are more robust to model misspecification than other longitudinal modeling approaches and do not require unverifiable assumptions [58]. The GEE models for weight loss reflected the binary outcome variable using a binomial distribution and logit link. To account for the dependency of repeated measures on individuals in a panel setting, we used an exchangeable correlation structure because we have no strong reasons to assume that the correlation between any two observations in the dataset might be stronger than the correlation between any two other observations [58–61]. The analysis estimated two sets of three additive models, including variables covering the social isolation index or the components of social isolation (Model 1), loneliness (Model 2), and depression (Model 3). All models were adjusted for other confounders and stratified by sex.

While the primary aim of this paper is to investigate the immediate impact of social isolation on a relatively sudden level of weight loss (within the past year), it is possible that social isolation could also have a long-term effect on changes in body weight. To investigate this cumulative effect, we conducted an ad hoc single-level logistic regression analysis using underweight status at the last observation as the dependent variable. Here, the cumulative effect among independent variables and confounders was measured as the average (for continuous variables, e.g., social isolation index, loneliness, and depression) or sum (for binary variables, e.g., living alone, widowhood status, divorce status, no frequent friend contact, no frequent child contact, and no frequent group activity) across all waves for a simple application. Thus, instead of the transition variable, the total number of observations of living alone, widowhood, or divorce, respectively, during the observation period were used. The (long-term) social isolation index was calculated by taking the mean of its relevant components. The analysis excluded individuals who were underweight at the start to remove pre-existing conditions. Stata 17 software was used for analysis, and the code is made available in S1 Appendix.

## Results

### Descriptive characteristics

Table 2 summarizes the sample’s descriptive characteristics at baseline among older men, stratified by living alone status. The analysis shows that men living alone and those living with someone else had a similar likelihood of experiencing weight loss of 5kg or more. While most characteristics were similar between individuals living alone and living with someone else, variables such as social isolation, marital status, loneliness, frequency of food consumption, and household income were found to differ between the two groups.

**Table 2:** Descriptive characteristics at baseline among men.

	Men living alone		Men living with someone else together		*P*-value
			someone else		
N of observations	153		2,229		
	% or mean	S.D.	% or mean	S.D.	
Weight loss of 5kg or more (%)	17.0		13.3		
Social isolation index (0–4)	1.9	0.07	0.9	0.02	***
Marital status (%)					
• Currently married	30.1		95.4		***
• Widowed	52.9		3.9		***
• Divorced / Separated / Not married	17.0		0.7		***
No friends contact within a month (%)	17.0		17.0		
No children contact within a month (%)	41.2		38.9		
No group activities within a month (%)	32.0		34.8		
Loneliness (1–4)	1.7	0.07	1.5	0.02	**
Depression (1–4)	1.6	0.06	1.5	0.02	
Number of meals over two days (0–6)	5.7	0.06	5.9	0.01	***
Drinking (%)	51.0		50.4		
Smoking (%)	30.7		25.5		
Physical activity at least once a week (%)	34.0		40.1		
BMI (12.5–36.3)	22.9	0.2	22.8	0.1	
Type 2 diabetes (%)	22.9		19.7		
ADL scale (0–7)	0.2	0.08	0.3	0.03	
IADL scale (0–10)	0.5	0.2	0.8	0.1	
Age group (%)					
• 65–74	73.8		76.0		
• 75–84	22.9		20.5		
• 85 and older	3.3		3.5		
Education (%)					
• Lower than middle school graduate	41.8		43.4		
• Middle school graduate	20.3		17.5		
• High school graduate	27.5		26.1		
• College graduate or higher	10.5		13.0		
Working for pay (%)	37.9		38.2		
Annual household income (0-$300K, $)	14,475	1,160	19,537	357	***
Geographic location (%)					
• Rural	31.4		29.6		
• City	31.4		30.3		
• Metropolitan	37.2		40.1		

*Note*. Significance levels

*** p < 0.001

** p < 0.01

* p < 0.05

Older men who lived alone scored higher on the social isolation index compared to those who lived with someone else. Specifically, the social isolation index score was 1.9 for men who lived alone, while it was 0.9 for men living with someone else (p < 0.001), which is not surprising as ‘living alone’ is one of the components of the social isolation index. While a majority of those who lived alone were either widowed (52.9%) or divorced/separated/unmarried (17.0%), most of the men who lived with someone else were currently married (95.4%). However, 30.1% of men living alone still indicated that they were currently married. Men who lived alone reported higher levels of loneliness, with a mean loneliness score of 1.7 compared to 1.5 for men living with someone else (p < 0.001). On average, men who lived alone consumed fewer meals over two days (5.7 meals for men living alone vs. 5.9 meals for men living with someone else, p < 0.001). Unsurprisingly, the average household income was lower for men living alone ($14,475) compared to men living with someone else ($19,537, p = 0.001).

Table 3 describes older women stratified by whether they lived alone or not. Despite having similar frequencies of weight loss of 5kg or more, most variables present significant differences between the individuals living alone or not (these include social isolation, marital status, friend contact, loneliness, depression, food consumption, ADL, IADL, age group, education, household income, and geographic location).

**Table 3:** Descriptive characteristics at baseline among women.

	Women living alone		Women living together		*P*-value
	alone		with		
			someone else		
N of observations	686		2,413		
	% or mean	S.D.	% or mean	S.D.	
Weight loss of 5kg or more (%)	12.7		11.9		
Social isolation index (0–4)	1.9	0.03	1.0	0.02	***
Marital status (%)					
• Currently married	6.9		68.8		***
• Widowed	90.2		30.4		***
• Divorced / Separated / Not married	2.9		0.07		**
No friends contact within a month (%)	12.5		17.3		**
No children contact within a month (%)	37.8		41.8		
No group activities within a month (%)	38.8		37.8		
Loneliness (1–4)	1.9	0.04	1.6	0.02	***
Depression (1–4)	1.8	0.03	1.7	0.02	***
Number of meals over two days (0–6)	5.7	0.03	5.9	0.01	***
Drinking (%)	13.8		11.2		
Smoking (%)	4.1		2.7		
Physical activity at least once a week (%)	26.8		28.5		
BMI (12.5–36.3)	23.1	0.1	23.2	0.1	
Type 2 diabetes (%)	20.6		19.6		
ADL scale (0–7)	0.2	0.03	0.3	0.02	**
IADL scale (0–10)	0.5	0.07	0.9	0.05	***
Age group (%)					
• 65–74	65.0		72.2		***
• 75–84	29.2		21.3		***
• 85 and older	5.8		6.5		
Education (%)					
• Lower than middle school graduate	82.9		75.8		***
• Middle school graduate	8.9		12.4		**
• High school graduate	6.1		9.8		***
• College graduate or higher	2.0		2.0		
Working for pay (%)	18.5		17.3		
Annual household income (0-$300K, $)	8,553	477	20,805	470	***
Geographic location (%)					
• Rural	32.4		28.5		
• City	26.2		31.1		*
• Metropolitan	41.4		40.4		

*Note*. Significance levels

*** p < 0.001

** p < 0.01

* p < 0.05

Briefly, older women who live alone have a greater social isolation index score compared to those who live with someone else (p < 0.001), similar to men. The majority of women living alone reported being widowed (90.2%), while those living with someone else included a substantial percentage of widows (30.4%). Most women who lived with someone else lived with a spouse (68.8%). Women living with someone else reported meeting friends less frequently (17.3% didn’t meet once a month), compared to women living alone (12.5%; p < 0.001). Despite this, women living alone reported higher average scores of loneliness (1.9) and depression (1.8) than those living with someone else (1.6 and 1.7, respectively). The average number of meals (out of a total of six over two days) consumed by women living alone was 5.7, while for women living with someone else, it was 5.9. These differences were statistically significant (p-values < 0.001), with many additional confounders differing as well.

### Prospective associations between social isolation and ≥5kg weight loss (GEE models)

Tables 4 (men) and 5 (women) show the odds ratios describing the relationship between social isolation (Models 1–3) / its components (Models 4–6) and weight loss of 5kg or more among older Koreans. In each table, Models 1 and 4 present the association between the social isolation index / its components and ≥5kg weight loss, while controlling for confounding variables, excluding loneliness and depression. Models 2 and 5 add loneliness as the first key confounder, and Models 3 and 6 include depression as the second key confounder.

**Table 4:** Estimated associations of weight loss of 5kg or more among men.

	Weight loss of 5kg or more, *Odds Ratios (CI)*					
	Social isolation approach			The component approach		
VARIABLES	Model 1	Model 2	Model 3	Model 4	Model 5	Model 6
Social isolation index (0–4)	1.10**	1.05	1.04			
	(1.02–1.17)	(0.98–1.13)	(0.97–1.11)			
Living alone & Marital status						
(ref. Continued living together without a change in marital status among the currently married)						
Continued living together without a change in marital status among widowers/divorcees				0.89	0.85	0.86
				(0.60–1.31)	(0.58–1.25)	(0.59–1.27)
Continued living alone without a change in marital status				1.07	0.96	0.97
				(0.77–1.51)	(0.69–1.34)	(0.70–1.34)
Transitioned to living alone after widowhood/divorce				1.02	0.92	0.90
				(0.52–2.01)	(0.48–1.79)	(0.47–1.73)
Continued living together after widowhood/divorce				1.49	1.35	1.34
				(0.79–2.84)	(0.72–2.54)	(0.71–2.52)
Transitioned to living together without a change in marital status				1.17	1.12	1.11
				(0.85–1.60)	(0.82–1.53)	(0.81–1.51)
Transitioned to living alone without a change in marital status				1.43*	1.39*	1.40*
				(1.08–1.90)	(1.05–1.84)	(1.05–1.85)
No friends contact within a month				1.32**	1.21*	1.17
(ref. Monthly contact with friends)				(1.11–1.58)	(1.01–1.45)	(0.98–1.40)
No children contact within a month				0.95	0.94	0.93
(ref. Monthly contact with children)				(0.83–1.08)	(0.82–1.06)	(0.82–1.06)
No group activities within a month				1.02	1.01	0.99
(ref. Monthly group activities)				(0.88–1.20)	(0.86–1.17)	(0.85–1.16)
Loneliness (1–4)		1.36***	1.17**		1.35***	1.16**
		(1.26–1.47)	(1.06–1.29)		(1.25–1.46)	(1.06–1.28)
Depression (1–4)			1.30***			1.29***
			(1.18–1.43)			(1.17–1.43)
Observations	8,564	8,564	8,564	8,564	8,564	8,564
Number of respondents	2,382	2,382	2,382	2,382	2,382	2,382

*Note*: All panel models adjust for behaviors (number of meals in prior two days; drinking; smoking; physical activity), health and functional status (BMI, type 2 diabetes; ADL scale, IADL scale), sociodemographic factors (age; education; working; household income; geographic location), and indicators for panel year.

*** p < 0.001

** p < 0.01

* p < 0.05

**Table 5:** Estimated associations of weight loss of 5kg or more among women.

	Weight loss of 5kg or more, *Odds Ratios (CI)*					
	Social isolation approach			The component approach		
VARIABLES	Model 1	Model 2	Model 3	Model 4	Model 5	Model 6
Social isolation index (0–4)	1.06	1.03	1.02			
	(0.99–1.12)	(0.97–1.10)	(0.96–1.08)			
Living alone & Marital status						
(ref. Continued living together without a change in marital status among the currently married)						
Continued living together without a change in marital status among widows/divorcées				1.02	1.01	1.02
				(0.85–1.23)	(0.84–1.21)	(0.85–1.22)
Continued living alone without a change in marital status				1.01	0.97	0.98
				(0.84–1.21)	(0.81–1.16)	(0.81–1.17)
Transitioned to living alone after widowhood/divorce				1.72**	1.58**	1.55*
				(1.22–2.43)	(1.12–2.24)	(1.09–2.21)
Continued living together after widowhood/divorce				1.30	1.21	1.18
				(0.88–1.93)	(0.81–1.79)	(0.79–1.76)
Transitioned to living together without a change in marital status				0.94	0.92	0.91
				(0.74–1.19)	(0.72–1.17)	(0.72–1.17)
Transitioned to living alone without a change in marital status				0.92	0.90	0.90
				(0.73–1.16)	(0.71–1.13)	(0.71–1.14)
No friends contact within a month				1.21*	1.15	1.12
(ref. Monthly contact with friends)				(1.03–1.43)	(0.98–1.36)	(0.95–1.32)
No children contact within a month				0.97	0.97	0.96
(ref. Monthly contact with children)				(0.86–1.10)	(0.86–1.09)	(0.86–1.09)
No group activities within a month				1.03	1.01	1.00
(ref. Monthly group activities)				(0.90–1.18)	(0.89–1.16)	(0.88–1.15)
Loneliness (1–4)		1.25***	1.05		1.23***	1.05
		(1.17–1.33)	(0.97–1.14)		(1.15–1.32)	(0.96–1.14)
Depression (1–4)			1.32***			1.31***
			(1.21–1.43)			(1.20–1.42)
Observations	11,341	11,341	11,341	11,341	11,341	11,341
Number of respondents	3,099	3,099	3,099	3,099	3,099	3,099

*Note*: All panel models adjust for behaviors (number of meals in prior two days; drinking; smoking; physical activity), health and functional status (BMI, type 2 diabetes; ADL scale, IADL scale), sociodemographic factors (age; education; working; household income; geographic location), and indicators for panel year.

*** p < 0.001

** p < 0.01

* p < 0.05

Table 4, Model 1 reports that men who experienced greater social isolation tended to lose 5kg or more weight (OR: 1.10; 95% CI [1.02–1.17]) with various confounders. However, after adjusting for loneliness in Model 2, the significance of this association disappeared. In Model 3, after depression was added, social isolation still had no association with weight loss. Thus, the study did not establish evidence to support Hypothesis 1 (a positive association between social isolation and weight loss) among men.

The study found that men who transitioned to living alone without experiencing the loss of a partner were more likely to undergo weight loss of 5kg or greater after considering loneliness and depression, compared to men who were currently married and living together (OR: 1.40; 95% CI [1.05–1.85]). Additionally, having no contact with friends within a month was also identified as a risk factor for ≥5kg weight loss (OR: 1.21; 95% CI [1.01–1.45]). However, after taking depression into consideration, the significance of this relationship disappeared. In conclusion, the study also did not find consistent support for Hypothesis 2 (the relationship between living alone after spousal bereavement or divorce and weight loss) among men.

Meanwhile, loneliness was found to be a significant risk factor for ≥5kg weight loss (OR: 1.36; 95% CI [1.26–1.47]) in Model 2. It maintained its significance (OR: 1.17; 95% CI [1.06–1.29]), and depression was also identified as a risk factor for ≥5kg weight loss (OR: 1.30; 95% CI [1.18–1.43]) in Model 3. The significance of loneliness and depression remained in Models 5 and 6, where the social isolation index was replaced with its components (OR: 1.16; 95% CI [1.06–1.28] for loneliness and OR: 1.29; 95% CI [1.17–1.43] for depression).

The results presented in Table 5 indicate no association between the social isolation index and weight loss of 5kg or more in women (Models 1–3). Therefore, there was no evidence supporting Hypothesis 1 for women. In Model 4–6, the analysis found that women who previously lived together and came to live alone after their spouse passed away or after a divorce were more likely to experience weight loss. This was backed up by evidence showing that the odds ratio was 1.72 (95% CI [1.22–2.43]) while considering various factors. Furthermore, this relationship persisted even after considering factors such as loneliness and depression (OR: 1.55; 95% CI [1.09–2.21]), in support of Hypothesis 2.

The study found that women who continued living alone after being widowed or divorced did not show a significant difference in weight loss compared to those who continued living with their spouses. This suggests that living alone due to widowhood or divorce may only have a short-term effect on weight loss. However, as a robustness check, the study also considered the cumulative effect of social isolation over time (Table 6). Neither the social isolation index nor any of its components show a significant association with being underweight at the last observation. This analysis also found that not having any contact with friends for a month was associated with weight loss (OR: 1.21, 95% CI [1.03–1.43]), though this relationship disappeared after taking loneliness into account.

**Table 6:** Estimated associations of underweight at the last observation.

	Underweight at the last observation, *Odds Ratios (CI)*			
	Men		Women	
VARIABLES	Model 1	Model 2	Model 1	Model 2
Avg. Social isolation index (0–4)	1.00		1.15	
	(0.73–1.38)		(0.93–1.43)	
Sum of living alone periods (0–7)		1.07		0.97
		(0.74–1.55)		(0.84–1.12)
Sum of widowed periods (0–7)		0.86		1.11
		(0.65–1.14)		(0.99–1.24)
Sum of divorced periods (0–7)		0.95		0.95
		(0.66–1.36)		(0.63–1.43)
Sum of periods of no frequent friend contact (0–7)		1.01		1.02
		(0.83–1.22)		(0.85–1.21)
Sum of periods of no frequent child contact (0–7)		0.93		1.00
		(0.81–1.06)		(0.89–1.13)
Sum of periods of no frequent group activities (0–7)		1.10		0.98
		(0.95–1.28)		(0.86–1.12)
Avg. Loneliness (1–4)	1.71	1.77	0.42*	0.40**
	(0.85–3.44)	(0.87–3.58)	(0.21–0.81)	(0.20–0.79)
Avg. Depression (1–4)	1.09	1.04	2.38**	2.52**
	(0.55–2.19)	(0.52–2.09)	(1.24–4.59)	(1.29–4.93)
Observations	2,419	2,419	3,143	3,143

*Note*: All models adjust for behaviors (number of meals in prior two days; drinking; smoking; physical activity), health and functional status (BMI, type 2 diabetes; ADL scale; IADL scale), and sociodemographic factors (age and education at entry; rural residence; household income). Except for age and education, all variables were constructed as a sum or mean during observations.

*** p < 0.001

** p < 0.01

* p < 0.05

In Table 5, Model 2 shows a significant link between loneliness and weight loss (OR: 1.25; 95% CI [1.17–1.33]). This relationship disappears in Model 3 after accounting for depression. Depression was identified as a risk factor for ≥5kg weight loss (OR: 1.32; 95% CI [1.21–1.43]). The same pattern of loneliness and depression was observed in Models 4–6, including the components of the social isolation index. Model 4 shows a connection between loneliness and weight loss (OR: 1.23; 95% CI [1.05–1.32]), while Model 5 reveals the only relationship between depression and weight loss (OR: 1.31; 95% CI [1.20–1.42]). As shown in Table 6, depression, as an average over the observations, was also a risk factor for being underweight at the last observation among women (OR: 2.38; 95% CI [1.24–4.59] in Model 1 and OR: 2.52; 95% CI [1.29–4.93] in Model 2). It was found that loneliness was unexpectedly linked to a lower likelihood of being underweight among women (OR: 0.42; 95% CI [0.21–0.81] in Model 1 and OR: 0.40; 95% CI [0.20–0.79] in Model 2).

## Discussion

Although research has been conducted on the relationship between social isolation and malnutrition [62–65], the extent to which different aspects of social isolation contribute to dangerous levels of weight loss (which is sometimes linked with malnutrition, but sometimes not) has not been extensively studied. In this study, a panel data analysis that took into account loneliness and depression found that different types of transition to living alone were associated with ≥5kg weight loss in ways that varied by gender. These findings remained robust even after accounting for a range of behavioral, health, functional status, and other sociodemographic factors.

The study found no evidence of a link between social isolation and harmful weight loss in this sample. While there initially appeared to be a connection among men, this disappeared when loneliness was taken into account. Depression was identified as a significant risk factor for dangerous weight loss, along with loneliness. This result reinforces the utility of a broad and multi-dimensional approach to the analysis of social isolation measures alongside measures of social isolation, enabling measurement of the relative contributions of each.

The current study found that women who transitioned to living alone after losing their spouse were at a higher risk, even when accounting for loneliness and depression. Prior research has shown that the end of a marriage can lead to a decrease in BMI [8,31,32], and this study confirms that spousal bereavement can also have negative health effects on women, particularly when their spousal loss ends with the transition to live alone. However, this study did not find significant evidence that living alone without any changes in marital status (mainly among widows) was related to a weight loss of 5kg or greater. This is consistent with a recent review that found a weak relationship between living alone and frailty in longitudinal studies, though we note that frailty and weight loss of 5kg or greater are separate measurement constructs [9]. However, older Korean women who live alone are vulnerable to malnutrition due to their lower socioeconomic status [53,54]. It is possible that living alone may still be associated with weight loss, but not at a clinically dangerous level.

In contrast to a US study conducted by Umberson and colleagues [8], which suggested that the effect of transitioning to widowhood on weight loss increased over time, our study found that the effect of living alone after spousal loss decreased over time. Specifically, our analysis showed that transitioning to living alone after spousal loss was a significant factor for weight loss, while either continued living alone or continued living together without any changes in marital status, mainly among widows, was insignificant. Additionally, our ad hoc analysis revealed that social isolation and its components did not have a cumulative effect on being underweight in the older Korean population. These findings suggest that a dangerous level of weight loss due to spousal bereavement may be temporary and perhaps recoverable over time. It is essential to note, however, that trends in body weight, frailty, and obesity differ between Korean and American older adults, so cross-national comparisons should be made with caution.

This study also reveals that the association between weight loss and living alone after spousal bereavement or divorce was only evident in older women, and not in men. This finding is similar to that of a single-sex study conducted on women [31], but contradicts the results of other studies. It is typically expected that older men would experience this association, as per a longitudinal study on changes in health behaviors and marital status among men [32]. One possible explanation could be that these men experiencing a dangerous level of weight loss, which is a risk factor for mortality, are excluded from the survey due to death. However, a test of gender difference in mortality during the observed periods suggests that this is not the case. In fact, a higher proportion of older women (n = 889 / 53.1%) than older men (n = 785 / 46.7%) were among the deceased. Survival or path analysis may be usefully employed to further investigate the relationships between the transition to living alone after spousal bereavement/divorce, ≥5kg weight loss, and mortality.

Instead, the present study has found that men who transition to living alone without any changes in marital status may be at a higher risk for clinically risky weight loss. To the best of our knowledge, this relationship is understudied. It is possible that these men are widowers living with their adult children, who may provide care for their father. During the first transition from being married to widowhood, these children may take care of their father, who is typically considered vulnerable to food provision. On the contrary, during the second transition from living together to living alone, widowers may struggle to eat, leading to significant weight loss. However, the current study suggests that this adaptation period may not be long-lasting. Further research is necessary to test these hypothetical pathways.

There was not sufficient evidence to conclude that other objective measures of social isolation, such as having no contact with friends within a month, having no contact with children within a month, or not participating in group activities within a month, were independently associated with ≥5kg weight loss after adjusting for various confounders. Although this finding contradicts several closely related prior studies [10,40,44], it is consistent with a study by Gale and colleagues [43], which used similar models to demonstrate that loneliness rather than social contact was a factor for frailty after adjusting for covariates.

Meanwhile, it is noteworthy that loneliness, as an average over the observations, was associated with a lower likelihood of being underweight at the last observation among women. Loneliness may have a short-term effect on weight loss, which is a component of frailty. However, its long-term impact may be related to not being underweight. Perhaps, this could be a reverse causation. Various studies indicate that there is a connection between obesity and loneliness [66,67]. Further research is necessary to elucidate these intricate pathways.

### Limitations

This study has several limitations, one of which is related to body weight measurement. As body weight was self-reported by the respondents, there is a possibility of recall bias, but any significant weight loss of 5kg or more is likely to be remembered. Moreover, many Koreans, especially older adults, regularly visit public baths where they habitually measure their body weight. Additionally, the validity of self-reported height and weight in a Korean sample that included older adults has been previously illustrated [68]. Nonetheless, the use of clinically-measured anthropometric information in addition to measures aside from simple height and weight, such as waist circumference, visceral fat, and body composition, in a culturally-tailored manner would better align with current recommendations [69,70]. Furthermore, people tend to experience cognitive decline in later life, and the use of objective measures can help ensure accurate data.

Another issue in the weight measurement is that the data does not provide information on whether the respondents intended to lose weight or not. It is possible that those who are overweight may participate in physical activities more frequently, possibly with someone else, to reduce their body weight. In this case, social isolation would be inversely related to weight loss. However, an additional analysis testing an association between overweight/obesity and the frequency of physical activities/social contact did not support this idea.

It also should be noted that this study documents but cannot provide a behavioral explanation for the relationship between living alone, the absence of a spouse, and weight loss in women. While previous research has explored the changes in eating habits following the loss of a spouse [37,71], the current study did not find any association between the number of meals taken in the last two days and ≥5kg weight loss among women. To gain a better understanding of this relationship, future research might focus on accounting for nutrient intake and reasons for missing meals. The composition and volume of each meal would be more important than simply not missing three meals a day, especially in light of the recent trend of intermittent fasting. In addition, while we considered it a strength to be able to investigate different categories of living alone and marital status, among men there were relatively few observations of older adults who continued to live together without a change in marital status, and those who transitioned to living alone without changing marital status.

Last, the present study was unable to take into account the experiences of social support and social contact due to a lack of information collected by KLoSA. These limitations could shed light on the counterintuitive insignificance of social contact in analyses. As prior research shows, social support often clearly plays a mediating role in the relationship between social contact and adverse health outcomes [39,41]. Several studies have also shown the importance of social support in preventing malnutrition [72–74]. Therefore, it is crucial to consider social support and social contact contexts to specify their roles.

## Conclusion

Our findings suggest that researchers and professionals working in the field of aging should pay special attention to the impact of a transition to living alone for women who have recently lost their spouse, and for men who maintain their marital status, with respect to preventing clinically-risky weight loss. In seeking accuracy, researchers should exercise caution while considering social isolation as a risk factor for adverse health outcomes, such as 5kg or more weight loss in a year, in population studies of health and aging. Such investigations can be more comprehensive by examining multiple forms of social isolation, loneliness, and depression, and also by comparing how the relationship may differ between men and women.

## Supporting information

S1 Appendix Sample code. (PDF)

S2 Appendix Minimal data set. (XLS)
