# Supplementary material for: Social isolation and depression as risk factors for weight loss of 5kg or more among older Korean adults
Source: PLoS One. 2024 Mar 13;19(3):e0299096. doi: 10.1371/journal.pone.0299096 (PMC10936863; doi:10.1371/journal.pone.0299096)
Supplement: S1 Appendix — (PDF) [file pone.0299096.s001.pdf]

Sample code

This is a sample code from the regression models (Stata Version 17) presented in our manuscript. This code is not exhaustive and does not include code for data cleaning or descriptive analysis. The data is available on the KLoSA website (https://survey.keis.or.kr/eng/klosa/klosa01.jsp). Please contact the corresponding author for questions or clarifications about analytic procedures.

* Caution: The variables' names were recoded and renamed from the original KLoSA variable names.

Tables 4 and 5

```
forvalue i=0/1 {

// Model 1: social isolation
xtgee weightloss_5kg ///
	new_isolaiton ///
	bmi alcohol smoke_2 meals6 diabetes ///
	iadl adl weekly_workout ///
	ib2.age_group labor i.edu i.area hhinc ///
	i.wave ///
	if new_isolaiton!=. & single_marital!=. & ///
	depression!=. & loneliness!=. & ///
	no_month_friends!=. & no_month_kids!=. & no_participation_month2!=. & ///
	bmi!=. & alcohol!=. & smoke_2!=. & meals6!=. & diabetes!=. & ///
	iadl!=. & adl!=. & weekly_workout!=. &  ///
	age_group!=. & labor!=. & edu!=. & area!=. & hhinc!=. & ///
	wave!=. & weightloss_5kg!=. &  ///
	female==`i' & age>64, i(pid) family(binomial) link(logit) corr(exc) vce(robust)

// Model 2: + loneliness
xtgee weightloss_5kg ///
	new_isolaiton ///
	loneliness ///
	bmi alcohol smoke_2 meals6 diabetes ///
	iadl adl weekly_workout ///
	ib2.age_group labor i.edu i.area hhinc ///
	i.wave ///
	if new_isolaiton!=. & single_marital!=. & ///
	depression!=. & loneliness!=. & ///
	no_month_friends!=. & no_month_kids!=. & no_participation_month2!=. & ///
	bmi!=. & alcohol!=. & smoke_2!=. & meals6!=. & diabetes!=. & ///
	iadl!=. & adl!=. & weekly_workout!=. &  ///
	age_group!=. & labor!=. & edu!=. & area!=. & hhinc!=. & ///
	wave!=. & weightloss_5kg!=. &  ///
```

```
    female==`i' & age>64, i(pid) family(binomial) link(logit) corr(exc) vce(robust)

// Model 3: + depression
xtgee weightloss_5kg ///
    new_isolaiton ///
    loneliness ///
    depression ///
    bmi alcohol smoke_2 meals6 diabetes ///
    iadl adl weekly_workout ///
    ib2.age_group labor i.edu i.area hhinc ///
    i.wave ///
    if new_isolaiton!=. & single_marital!=. & ///
    depression!=. & loneliness!=. & ///
    no_month_friends!=. & no_month_kids!=. & no_participation_month2!=. & ///
    bmi!=. & alcohol!=. & smoke_2!=. & meals6!=. & diabetes!=. & ///
    iadl!=. & adl!=. & weekly_workout!=. &  ///
    age_group!=. & labor!=. & edu!=. & area!=. & hhinc!=. & ///
    wave!=. & weightloss_5kg!=. &  ///
    female==`i' & age>64, i(pid) family(binomial) link(logit) corr(exc) vce(robust)
}

forvalue i=0/1 {
// Model 4: components of social isolation
xtgee weightloss_5kg ///
    i.single_marital2 ///
    no_month_friends no_month_kids no_participation_month2 ///
    bmi alcohol smoke_2 meals6 diabetes ///
    iadl adl weekly_workout ///
    ib2.age_group labor i.edu i.area hhinc ///
    i.wave ///
    if new_isolation!=. & single_marital!=. & ///
    depression!=. & loneliness!=. & ///
    no_month_friends!=. & no_month_kids!=. & no_participation_month2!=. & ///
    bmi!=. & alcohol!=. & smoke_2!=. & meals6!=. & diabetes!=. & ///
    iadl!=. & adl!=. & weekly_workout!=. &  ///
    age_group!=. & labor!=. & edu!=. & area!=. & hhinc!=. & ///
    wave!=. & weightloss_5kg!=. &  ///
    female==`i' & age>64, i(pid) family(binomial) link(logit) corr(exc) vce(robust)

// Model 5: + loneliness
xtgee weightloss_5kg ///
    i.single_marital2 ///
    no_month_friends no_month_kids no_participation_month2 ///
    loneliness ///
    bmi alcohol smoke_2 meals6 diabetes ///
    iadl adl weekly_workout ///
```

```
        ib2.age_group labor i.edu i.area hhinc ///
        i.wave ///
        if new_isolation!=. & single_marital!=. & ///
        depression!=. & loneliness!=. & ///
        no_month_friends!=. & no_month_kids!=. & no_participation_month2!=. & ///
        bmi!=. & alcohol!=. & smoke_2!=. & meals6!=. & diabetes!=. & ///
        iadl!=. & adl!=. & weekly_workout!=. &  ///
        age_group!=. & labor!=. & edu!=. & area!=. & hhinc!=. & ///
        wave!=. & weightloss_5kg!=. &  ///
        female==`i' & age>64, i(pid) family(binomial) link(logit) corr(exc) vce(robust)

// Model 6: + depression
xtgee weightloss_5kg ///
        i.single_marital2 ///
        no_month_friends no_month_kids no_participation_month2 ///
        loneliness ///
        depression ///
        bmi alcohol smoke_2 meals6 diabetes ///
        iadl adl weekly_workout ///
        ib2.age_group labor i.edu i.area hhinc ///
        i.wave ///
        if new_isolation!=. & single_marital2!=. & ///
        depression!=. & loneliness!=. & ///
        no_month_friends!=. & no_month_kids!=. & no_participation_month2!=. & ///
        bmi!=. & alcohol!=. & smoke_2!=. & meals6!=. & diabetes!=. & ///
        iadl!=. & adl!=. & weekly_workout!=. &  ///
        age_group!=. & labor!=. & edu!=. & area!=. & hhinc!=. & ///
        wave!=. & weightloss_5kg!=. & ///
        female==`i' & age>64, i(pid) family(binomial) link(logit) corr(exc) vce(robust)
}
```
